# Kidney-disease-associated variants of Apolipoprotein L1 show gain of function in cation channel activity

**DOI:** 10.1074/jbc.RA120.013943

**Published:** 2021-01-09

**Authors:** Jonathan Bruno, John C. Edwards

**Affiliations:** Nephrology Division, Department of Internal Medicine, Saint Louis University, St Louis, Missouri, USA

**Keywords:** ApoL1, apolipoprotein, cation channel, chloride channel, ion channel, phospholipid vesicle, potassium channel, protein–lipid interaction, FSGS, kidney, ApoL1, apolipoprotein L1, CI1, chloride ionophore 1, DDM, n-dodecyl-β-D-maltoside, PVDF, polyvinylidene fluoride, SD, standard deviation, Val, valinomycin

## Abstract

Variants in Apolipoprotein L1 (ApoL1) are known to be responsible for increased risk of some progressive kidney diseases among people of African ancestry. ApoL1 is an amphitropic protein that can insert into phospholipid membranes and confer anion- or cation-selective permeability to phospholipid membranes depending on pH. Whether these activities differ among the variants or whether they contribute to disease pathogenesis is unknown. We used assays of voltage-driven ion flux from phospholipid vesicles and of stable membrane association to assess differences among ApoL1 isoforms. There is a significant (approximately twofold) increase in the cation-selective ion permease activity of the two kidney-disease-associated variants compared with the reference protein. In contrast, we find no difference in the anion-selective permease activity at low pH among the isoforms. Compared with the reference sequence, the two disease-associated variants show increased stable association with phospholipid vesicles under conditions that support the cation permease activity, suggesting that the increased activity may be due to more efficient membrane association and insertion. There is no difference in membrane association among isoforms under optimal conditions for the anion permease activity. These data support a model in which enhanced cation permeability may contribute to the progressive kidney diseases associated with high-risk ApoL1 alleles.

Two distinct variants in the protein ApoL1 are known to be responsible for the recognized increased risk of progressive proteinuric kidney disease in people of African ancestry, particularly due to focal segmental glomerulosclerosis, hypertensive nephrosclerosis, and HIV-associated nephropathy (1, 2, 3). The functional roles of ApoL1 in human cells, the impact of the disease associated variants on these functions, and how any such alterations in activity may accelerate kidney disease are unclear (4, 5).

The only biochemical activity that the ApoL1 protein is known to possess is to function as an ion channel. The N-terminal domain of ApoL1 has been noted to have structural similarity to bacterial colicin A1 (6). Like colicin A1, ApoL1 is an amphitropic protein that under certain circumstances can enter a lipid membrane from aqueous solution and then function as an ion permease (6, 7, 8, 9). Purified recombinant wild-type ApoL1 is stable in detergent solution at neutral pH (8, 9). At low pH, ApoL1 can enter phospholipid membranes and increase the anion-selective permeability of the membranes (6, 7, 9). When cis pH is raised back to neutral, the anion-selective permeability is suppressed and a cation-selective channel is activated (8, 9). These data support a model in which ApoL1, presented to an acidic compartment along either the exocytic or endocytic pathways, could insert into the organelle’s limiting membrane leading to increased anion permeability. Subsequent trafficking of the protein to other membrane compartments where the cis pH would be neutral (such as the plasma membrane or mitochondrial membranes) would suppress the anion permeability and activate the cation permease. Whether these ion permease activities play a role in pathogenesis of the ApoL1-associated renal diseases is unclear.

We used vesicle-based methods to assay for differences in ion permease activities and membrane binding between the reference (wild type) sequence, designated G0, and the two disease-associated variants, designated G1 and G2. We find that compared with G0, both G1 and G2 isoforms show increased specific activity of the cation permease activity at neutral pH without altering the anion-selective permease activity at low pH. Further, the disease-associated isoforms G1 and G2 show increased stable membrane association compared with G0 under conditions that support the cation permease activity but not under conditions that support the anion permease activity. We do not find any effect on the pattern of pH sensitivity of either the binding step or the efflux step of the cation channel activity, and we find no effect on the pH sensitivity of the anion-selective permease activity. We conclude that the disease-associated variants of ApoL1 selectively enhance the cation channel activity of the protein, likely at least partially by enhancing membrane insertion. We propose that this enhanced channel activity contributes to progressive kidney disease in people carrying high-risk genotypes.

## Results

**Figure 1:**
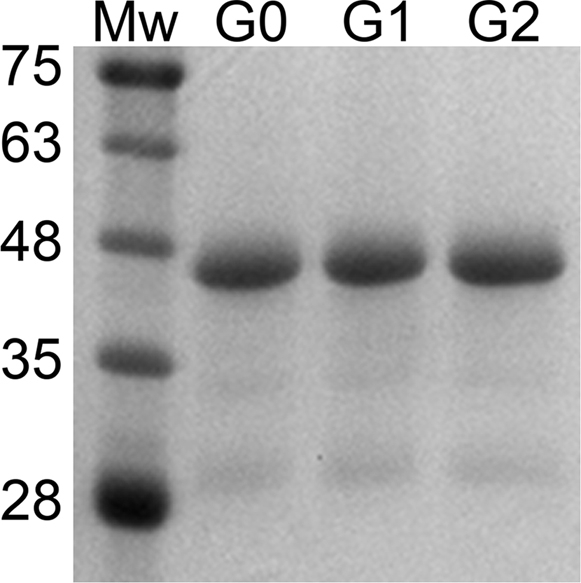
**Purified ApoL1 preparations.** Molecular size markers (Mw) and 1 μg of purified recombinant ApoL1 isoforms G0, G1, and G2 were separated by SDS-PAGE and stained for protein. Markers are labeled with the molecular weight in kiloDaltons.

Recombinant isoforms of ApoL1 with the N-terminal signal sequence removed and replaced with a 6His/T7 epitope tag were expressed and purified. All three showed identical chromatographic properties through the purification and were indistinguishable by gel electrophoresis (Fig. 1).

Peak fractions were diluted to 100 μg/ml and subjected to vesicle efflux assays for ability to confer chloride and potassium permeability. The assay consists of two separate stages. In the association stage, ApoL1 is mixed with KCl-loaded phospholipid vesicles and allowed to insert into the membranes. Extravesicular KCl and buffer are then removed by passage through a desalting spin column equilibrated in isotonic sucrose, generating large inside-to-outside gradients of both K and Cl ions. In the efflux stage, the spin column eluate is diluted into an isotonic buffered sucrose solution that maintains the KCl gradient at the desired pH. Extravesicular Cl concentration is monitored with a chloride-selective electrode. Even if either K or Cl-selective permeability is present, neither ion will be able to exit the vesicles because of lack of counterion movement. Voltage-driven ion efflux is initiated by addition of a saturating concentration of a counterion ionophore. Addition of the K-selective ionophore, valinomycin (Val), will induce a large inside-negative membrane potential that, if Cl permeability is present, will drive KCl efflux that will be detected by the chloride-selective electrode; thus after Val, the rate of Cl efflux reflects Cl permeability. Conversely, addition of the Cl-selective ionophore, Chloride Ionophore 1 (CI1), will induce an inside-positive membrane potential that will drive KCl efflux if a K permeability is present. Hence, after addition of CI1, the rate of Cl efflux reflects K permeability. This assay thus can serve as either a Cl or K permeability assay, depending on which ionophore is used. The initial rate of ion efflux following addition of ionophore is taken as the specific ionic permeability of the vesicles. Note, Cl efflux prior to addition of ionophore indicates some permeability to both K and Cl. Even in this circumstance, the rate of Cl efflux after addition of an excess of the counterion ionophore represents the permeability to the ion of interest—Cl after addition of Val, and K after addition of CI1.

### Chloride permease activity

**Figure 2:**
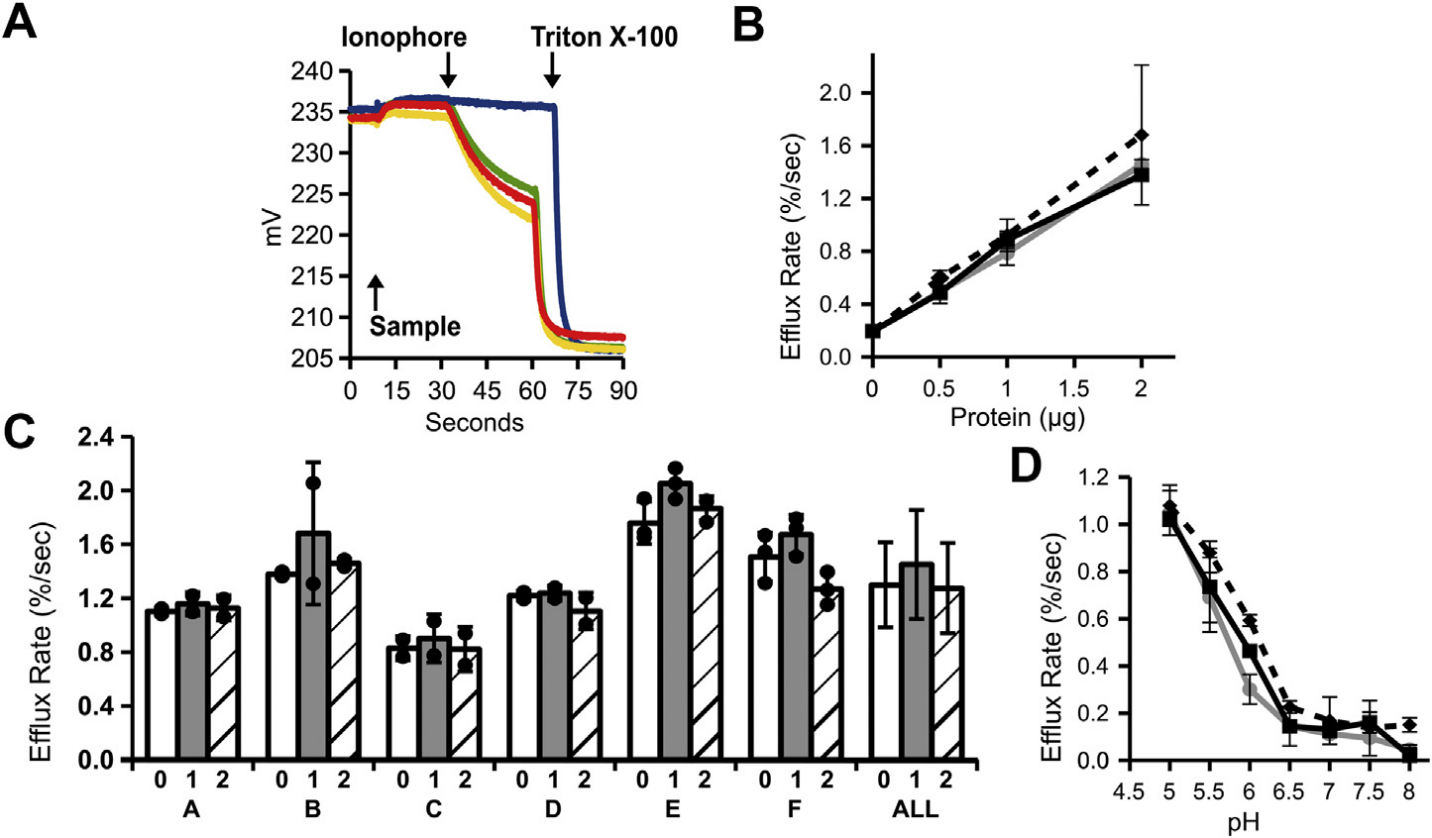
**Cl permease activity of ApoL1 isoforms**. *A*, raw data from representative Cl permease assays using 2 μg of each isoform. The output of chloride selective electrode in mV is plotted over time. *First arrow* marks addition of the spin column eluate. *Second arrow* marks addition of Val. *Third arrow* marks addition of Triton X-100. *Blue tracing*: control (no protein); *Red*: G0; *Yellow*: G1; *Green*: G2. *B*, 0, 0.5, 1.0, or 2.0 μg of purified ApoL1 isoforms were assayed for anion permease activity at pH 5. Fractional release of Cl (%/s) after addition of Val is plotted *versus* mass of ApoL1. *Square marker with solid line*: G0; *diamond marker with dashed line*: G1; *circle marker with gray line*: G2. Means ± SD are plotted, n = 2 for each data point. There was no significant difference of rates between isoforms at any concentration. *C*, comparison of data from six independent sets of protein preparations (A–F) and average of all preparations (ALL). In each set, rates of efflux supported by 2.0 μg of G0, G1, and G2 isoforms are shown as marked. Individual data points are represented by filled circles. The height of the bar graph represents the mean, the error bars represent SD. N = 2 for each group in sets A–D, N = 3 for each group in sets E, F. Mean values from the six sets were averaged to produce the “ALL” set. There were no significant differences in rates among the isoforms within any set. *D*, 2.0 μg of each ApoL1 isoform was assayed for anion permeability at pH values indicated. *Markers/lines* as in *B*. Means ± SD are plotted, n = 2 for each data point.

Our previous studies showed that the anion permeability is greatest when both the membrane association step and the efflux step are carried out at pH 5.0, and these conditions were used to compare Cl permeability activity of the three isoforms, using Val to initiate Cl permeability-dependent efflux. Examples of raw data are shown in Figure 2*A*, which shows the output from the Cl-selective electrode in millivolts plotted *versus* time. The recording begins prior to addition of the spin column eluate containing vesicles and protein, the mV value reflecting the 10 μM Cl concentration. The Cl-free spin column eluate is added at the first arrow, diluting the Cl, which is reflected in the upward deflection of the mV tracing. Val is added at the second arrow, triggering Cl efflux if Cl permeability is present, which is reflected by the progressive drop in mV. Triton X-100 is added at the third arrow, releasing all remaining intravesicular chloride. After conversion from mV to chloride concentration using a standard curve generated for the electrode under conditions of the assay, the fractional initial rate of Cl release after addition of Val is determined and taken as the chloride permeability. Compared with the no-protein control (blue tracing), the high rate of Cl release after addition of Val indicates that ApoL1 confers substantial Cl permeability that appears comparable among the three isoforms (red, green, and yellow tracings). The very low rate of efflux prior to Val indicates that the potassium permeability is much lower than the Cl permeability. A range of concentrations of the ApoL1 isoforms were assayed for Cl permease activity (Fig. 2*B*). As previously reported for G0, we find Cl permease activity is linearly related to mass of protein in the assay for all three isoforms. The permease activity at each concentration is no different among the three isoforms. The concentration curve was performed twice using two different sets of protein preparations, yielding the same result.

Six independent preparations of the three proteins were assayed for Cl permease activity at pH 5.0 as summarized in Figure 2*C*. In none of the sets of preparations did we find a significant difference in Cl permease activity among the isoforms (*p* > 0.05 for comparison of G0 with G1 or G2 in each set). The average efflux rates of all six preparations (labelled ALL in Fig. 2*C*) were 1.21 ± 0.32 %/s for the reference sequence G0, 1.36 ± 0.41 %/s for G1, and 1.18 ± 0.34 for G2 (mean ± SD, n = 6 for each; *p* = 0.47 and 0.94 for comparison of G0 with G1 and G2, respectively). To determine whether the disease-associated mutations alter the pH sensitivity of the anion permease, Cl permeability conferred by each isoform was assayed through a range of pH values, using the indicated pH for both association and efflux steps of the assay (Fig. 2*D*). We found no significant differences among the isoforms. This experiment was performed twice using two different sets of protein preparations, yielding the same result. Thus, the Cl permease activity is not significantly different between the two disease-associated variants and the reference sequence.

### Potassium permease activity

**Figure 3:**
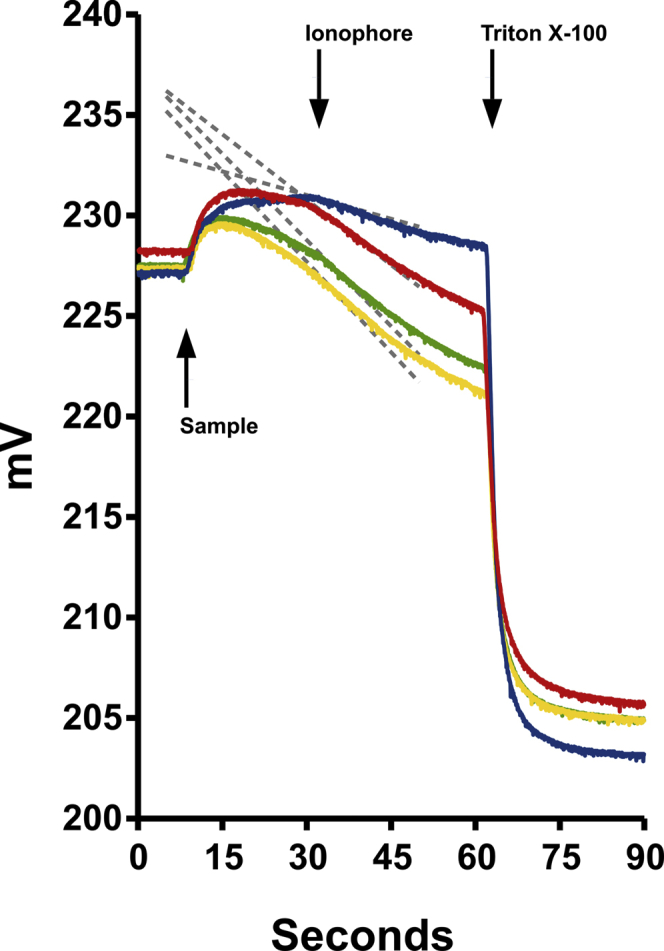
**Raw data of representative K permease assays.** Plot as in Figure 2*A*, except *second arrow* marks addition of CI1. *Blue tracing*: control (no protein); *red*: G0; *yellow*: G1; *green*: G2. The *dotted lines* represent the initial rates of change after addition of ionophore. Note the difference in rates between G0 and G1/G2 samples.

Our previous studies indicated that cation permeability is greatest when the membrane association step is carried out at pH 6.0 and the efflux step is carried out at pH 7.5, and we used these conditions unless specified otherwise. Examples of raw data from a K permease assay are shown in Figure 3. As with the Cl permeability assays, the spin column eluate is added at the first arrow, the ionophore (in this case, Chloride Ionophore 1, CI1) is added at the second arrow, and detergent is added at the third arrow. The initial rates of efflux after addition of ionophore, representing the K permeability, are shown by the gray dashed lines. The blue tracing is from the no protein control, showing the endogenous background permeability of the vesicles alone. The red tracing shows activity of the G0 (wild type) variant, demonstrating clearly the presence of K permeability greater than control vesicles conferred by the protein as previously reported (9). The yellow and green traces show activity of the G1 and G2 variants, respectively. The initial slopes after addition of ionophore are clearly greater for the G1 and G2 than for G0, indicating that the disease-associated variants support greater K permease activity than does G0.

**Figure 4:**
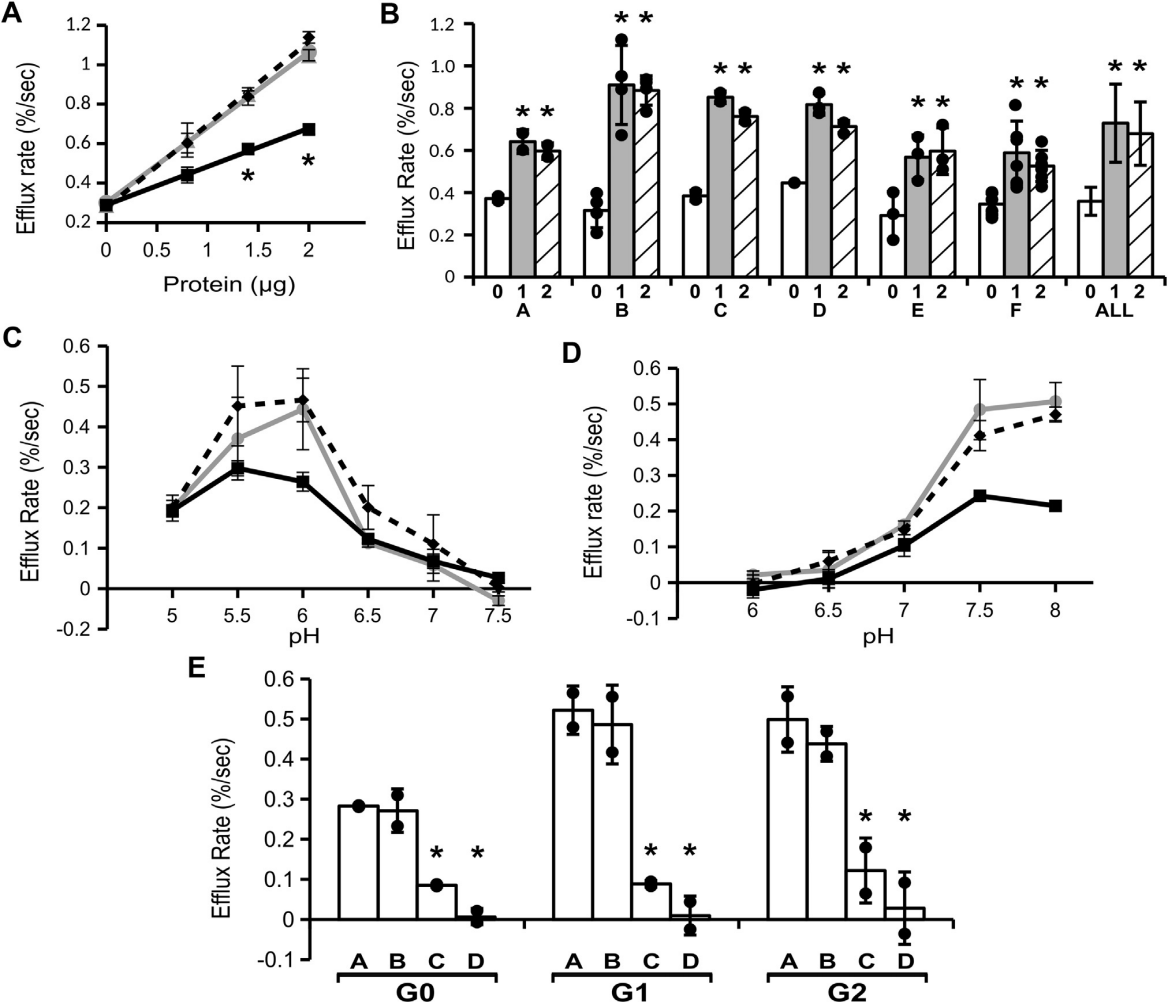
**Cation permease activity of ApoL1 isoforms. *A*,** 0, 0.8, 1.4, or 2.0 μg of purified ApoL1 isoforms were assayed for K permease activity with membrane association at pH 6.0 and efflux at pH 7.5. As in Figure 2*A*, *square marker with solid line*: G0; *diamond marker with dashed line*: G1; *circle marker with gray line*: G2. Means ± SD are plotted, n = 2 for each data point. *Asterisks* indicate points at which the G0 rate is significantly lower than both the G1/G2 rates (*p* < 0.05). *B*, activities from six independent sets of preparations (A–F) and all combined (All). In each set, rates of efflux supported by 2.0 μg of G0, G1, and G2 isoforms are shown as marked. Individual data points are shown as filled circles. The height of the bar graph represents the mean, the error bars represent SD. *Asterisks* indicate *p* < 0.05 compared with G0 in each set. N = 2 for each group in sets A and C, 3 for each group in sets D and E, 4 in each group in set B, and 6 in each group in set F. Mean values from the six sets were averaged to produce the “ALL” set. *C*, pH sensitivity of membrane association step. Purified ApoL1 isoforms (2.0 μg each) were mixed with vesicles at the pH values as marked and efflux rates determined after addition of chloride ionophore at pH 7.5. Each data point represents the mean ± SD, n = 2 for each. *Marker/lines* indicate isoforms as in panel *A*. *D*, pH sensitivity of efflux step. Purified ApoL1 isoforms (2.0 μg each) were mixed with vesicles at pH 6.0 and efflux rates determined at the pH values as marked. Each data point represents the mean ± SD, N = 2 for each. *Marker/lines* indicate isoforms as in panel *A*. *E*, dependence on divalent cation and intravesicular pH. Isoforms (2.0 μg each) were assayed for K permease activity under (*A*) standard conditions; (*B*) with magnesium substituted for calcium throughout the assay; (*C*) in the absence of divalent cation throughout the assay; or (*D*) in the presence of calcium but with vesicles in which internal pH was buffered at pH 5. Individual data points for efflux rate above the no-protein control rate under each condition are shown, height of bar graph represents mean, error bars represent SD, N = 2 for each, *asterisks* denote *p* < 0.05 compared with the standard conditions value in each set.

In contrast to the Cl permeability assay, and as previously reported for the G0 isoform (9), under the K permease assay conditions, there is some efflux prior to ionophore in the vesicles containing any of the three isoforms, represented by the downward slope of the recordings after addition of sample and before addition of ionophore. While the high rate of efflux after addition of ionophore indicates the presence of substantial K permeability, efflux prior to ionophore indicates the presence of some Cl permeability as well, allowing both ions to leak out of the vesicles in the absence of ionophore. This leak likely accounts for both the blunted rise in electrode output immediately after addition of the spin column eluate and the subsequent downward slope prior to CI1 that is seen with all recordings containing ApoL1. Assuming this leak begins as the sample is passing through the spin column, it also would account for the differences in the final Cl concentration after addition of detergent, since any loss of KCl on the spin column would mean less total KCl added to the assay. The quantitative interpretation of the preionophore rate is not straightforward other than it must be a function of the permeabilities to both K and Cl. As before (9), we attribute this activity to the residual Cl permease activity that we see at pH 7.5 as in Figure 2*D* (see also Bruno *et al.* (9), Fig. 4*D*).

Potassium permease assays were carried out over a range of protein concentrations (Fig. 4*A*). The activity was linearly related to the mass of protein in the assay for each isoform, but the two disease-associated isoforms showed substantially increased efflux activity compared with G0. This concentration curve was reproduced with three separate sets of protein preparation yielding very similar results.

Six independent preparations of the three isoforms were assayed for K efflux activity using 2 μg of protein in each assay. In each of the six sets, activity for the G1 and G2 isoforms was significantly greater than that of G0 (Fig. 4*B*). Average of all six data sets yielded efflux rates of 0.349 ± 0.063 %/s for G0, 0.709 ± 0.176 %/s for G1, and 0.673 ± 0.142 %/s for G2 (mean ± SD, n = 6 for each; *p* < 0.00001 for comparison of G0 with either G1 or G2). The specific activities of the K permease of G1 and G2 are 2.03- and 1.93-fold greater, respectively, than G0 by this assay.

Dependence of the K permease activity on the pH of the membrane association step and the ion efflux step was analyzed separately. Although we confirmed that the total efflux activity of G1 and G2 is greater than that of G0 under “standard” conditions (binding at pH 6, efflux at pH 7.5), we found no significant difference in the shape of the curve of activity *versus* external (cis) pH of either the membrane association step (Fig. 4*C*) or the ion efflux step (Fig. 4*D*). We also found no differences among the isoforms in the dependence of activity on high intravesicular (trans) pH, or the necessity for presence of divalent cation (Fig. 4*E*), and further note that either calcium or magnesium is equally efficient in providing the necessary divalent cation for full activity. Each of these experiments was performed twice using different sets of purified protein, yielding identical results.

### Stable membrane association

**Figure 5:**
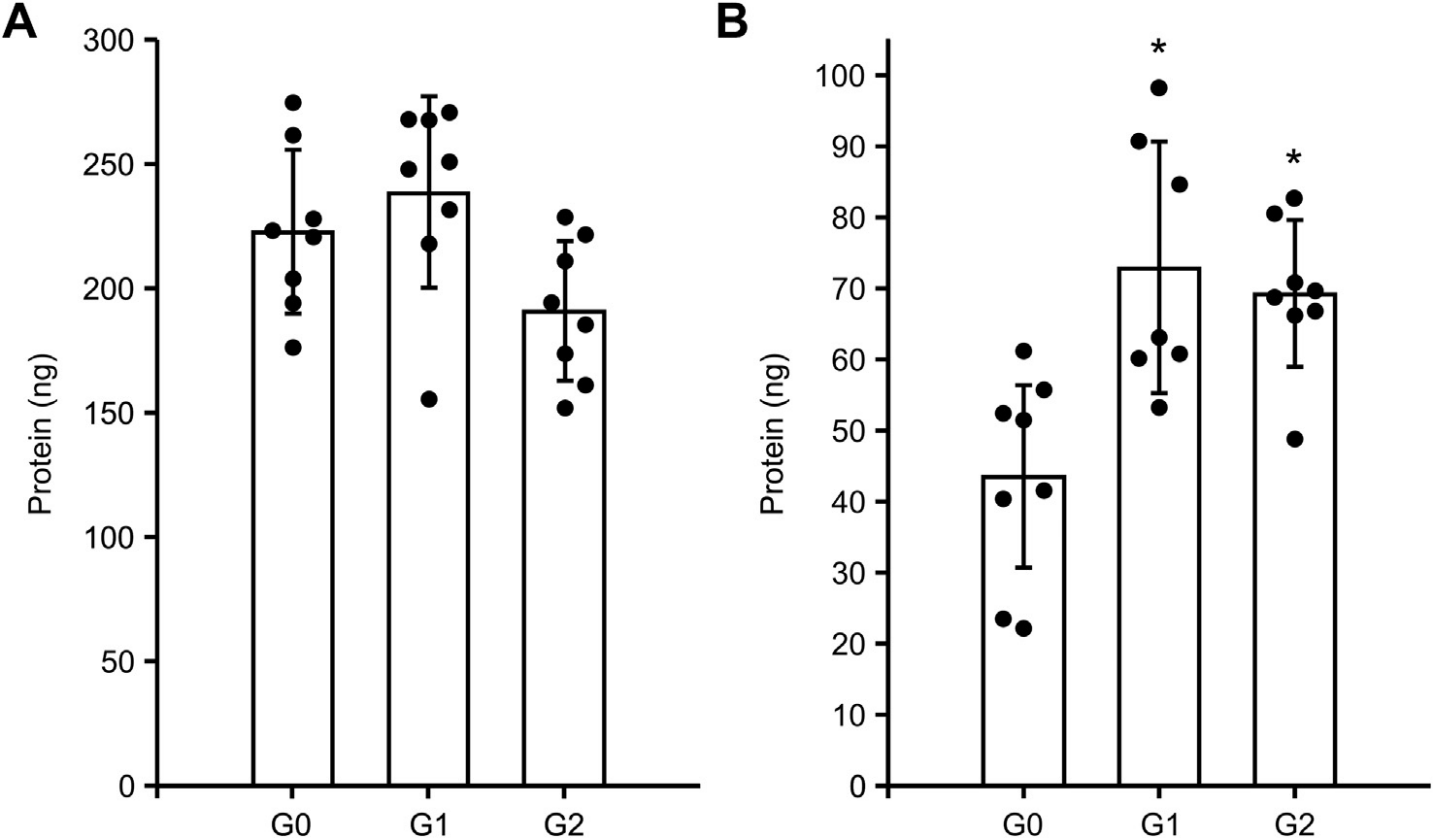
**Stable membrane association of ApoL1 isoforms under conditions supporting anion or cation permease activity.** Mass of protein stably associated with phospholipid vesicles after chaotropic extraction and flotation through sucrose cushion was determined by quantitative western blotting. Individual data points are plotted. Height of the bars graph represents the mean, error bars represents SD. *Asterisks* indicate *p* < 0.05 for comparison with G0. Note the difference in the scale of *y*-axis between panels *A* and *B*. *A*, assay performed under conditions optimal for anion permease activity. There is no significant difference between G0 and either G1 or G2. n = 8 for each group. *B*, assay performed under conditions optimal for cation permease activity. The amount bound is significantly greater for both disease-associated variants than for G0. n = 8 for G0 and G2, n = 7 for G1.

We have previously demonstrated that purified recombinant ApoL1 spontaneously binds to phospholipid membranes showing similar dependence on pH and phospholipid composition as that of the ion permease activities (9). Using similar methods, we compared the lipid vesicle binding properties of the reference version of ApoL1 with the kidney-disease-associated variants under conditions supporting optimal Cl or K permease activities (Fig. 5). For the Cl permease conditions, protein was incubated with lipid vesicles at pH 5.0 prior to chaotropic extraction to remove proteins that were not stably associated with the vesicles. Membrane-bound protein was isolated by flotation through a sucrose cushion and assessed by quantitative western blotting. Results are shown in Figure 5*A*. There was no significant difference in the amount of protein that bound to vesicles between G0 and either G1 or G2 isoforms. Of the 500 ng protein in each reaction, the amount bound to vesicles was 223 ± 33 ng for G0, 239 ± 38 ng for G1, and 191 ± 28 ng for G2 (mean ± SD; n = 8 for each group, *p* = 0.35 and 0.076 for comparison of G0 with G1 and G2, respectively). Approximately 40–50% of the protein in the assay bound to membranes under these conditions. This experiment was done twice yielding very similar results.

To assess binding under conditions optimal for the K permease activity, protein was incubated with vesicles at pH 6.0, then shifted to pH 7.5 prior to chaotropic extraction to remove nonstably inserted protein. Membrane-bound protein was isolated by flotation of the membrane vesicles through a sucrose cushion and assessed by quantitative western blotting. Results are shown in Figure 5*B*. The disease-associated variants show significantly more efficient binding compared with the G0. Of the 500 ng of protein added to each reaction, the amount bound to vesicles was 43.5 ± 14.5 ng (n = 8) for G0, 73.0 ± 17.7 ng (n = 7) for G1, and 69.3 ± 10.3 ng (n = 8) for G2 (mean ± SD; *p* = 0.00076 and 0.0018 for comparison of G0 with G1 and G2, respectively). Approximately 9% of the G0 protein in the reaction was stably associated with the vesicles compared with approximately 14% of the G1 and G2 under these conditions. This experiment was done twice using independent preparations yielding similar results.

## Discussion

We here present results of studies of ion permease activities of ApoL1 and its kidney-disease-associated variants that yielded several significant results. First, we find that the disease-associated variants of ApoL1 have substantially increased K permease activity, which is approximately double that of the G0 isoform in our assays. Second, in contrast to the K permease, there are no significant differences in the specific activity of the Cl permease activity at pH 5.0 among the isoforms. Third, there are no differences in the pH sensitivity of either the K or Cl permease activities, or the requirement for high trans pH and the necessity of divalent cation for the K permease activity among the isoforms. Fourth, under optimal conditions supporting the K permease, the disease-associated variants show enhanced stable membrane association compared with G0, but no difference in membrane association under optimal conditions for the Cl permease activity.

Our findings demonstrating increased K permease with no effect on Cl permease are very robust. Using optimal conditions for each permease activity, we found essentially identical results among six independent sets of preparations, with the K permease activity of the disease-associated variants significantly greater than G0 in each. Each set was prepared and assayed at the same time using common reagents. The validity of the protein concentrations determined by BCA assay was confirmed by comparable intensity of Coomassie staining on SDS-PAGE. These data include all successful attempts at simultaneous purification—we never successfully generated a set of all three variants in which these relative activities were not observed.

Likewise, the absence of differences in the anion-permease-specific activity at pH 5 was consistent among all the sets of preparations. Thus our primary conclusion is that the disease-associated variants of ApoL1 confer selective gain of function in the K permease activity without affecting the Cl permease activity, as assayed under optimal conditions for each of these activities. The presence of substantial differences in the cation channel activity of ApoL1 variants that correlates with capacity to drive disease has implications for possible pathogenic mechanisms. The selective nature of the increased ion permeability indicates that increased cation permease activity of the disease-associated variants could contribute to disease, but that the anion permease activity at low pH, which is not different among the isoforms, is unlikely to play a critical role.

The investigations of effects of the variants on the pH sensitivities of the activities and on the extent of stable membrane binding by the protein shed light on the mechanism by which the K permease activity may be enhanced. First, other than confirming the enhanced activity of the K permease under optimal conditions, we found no differences in the shapes of the curves showing pH effects on the membrane association and efflux steps of the K permease activity or of the effect of pH on overall Cl permease activity. Further, there were no differences in the requirement for elevated pH of the interior of the vesicles or in the requirement for the presence of divalent cation in the reaction for the K permease activity among all isoforms. Thus we found no evidence for difference in the intrinsic properties of the channel itself. In contrast, the variants showed significantly increased stable association with phospholipid vesicles under conditions that support the K permease activity, with the scale of this effect in roughly the same range as the change in activity. Taken together, the data suggest that the basis for the increased K permease activity is simply more efficient membrane insertion under the conditions supporting the activity, without the need to invoke a fundamental change in the properties of the inserted protein.

Three domains have been identified in the ApoL1 structure (6). The ion channel activity is thought to lie in the colicin-homologous “pore forming” domain, which occupies about 60% of the protein at its N terminus. The disease-associated sequence variations are not in the pore forming domain, but lie at a distance from it in a coiled-coil domain, which comprises about 16% of the protein at the C terminus. Deletion of this domain has no effect on trypanolytic activity but is highly conserved among ApoL isoforms both within and between species (10). It has been suggested this region functions as a control domain (10), and our data indicate that disease-causing mutants in this domain do indeed have increased membrane binding under conditions that support cation channel activity and increased cation channel activity. One interpretation is that the C-terminal domain serves to suppress the membrane association/insertion necessary for cation permease activity, and mutation of this domain relaxes this suppression.

The observations on membrane binding have further implications and illuminate some aspects of the relationship between anion and cation permease activities. We here find that substantially more protein is bound to membranes at optimal anion permease activity conditions (pH 5.0) than at optimal binding conditions supporting cation permease activity (pH 6.0), and this is true for all isoforms (Fig. 5). Note the difference in the pH sensitivities of the anion permease activity (Fig. 2*D*) and the membrane binding step of the cation permease activity (Fig. 4*C*): anion permease increases dramatically below pH 6, while the ability of protein inserted into the membrane to support cation permease activity drops significantly. Our previously reported data showed that stable membrane association of G0 rises substantially as pH falls through that same pH range, and that is the range where intrinsic protein fluorescence is most dramatically suppressed (9), indicating a significant transition in the structure of the protein. Taken all together the data suggests that membrane insertion at pH 6.0 is fundamentally different than membrane insertion at pH 5.0. At pH 6.0, a smaller fraction of the protein associates with membranes, accompanied by only minor change in intrinsic fluorescence and limited anion permease activity, but with the capacity to transition to a cation permeable form when the cis compartment is titrated to pH 7.5. Further, the efficiency of this binding is greater for the disease associated variants, paralleling the increased cation permease activity after pH shift. In contrast, at pH 5.0 a much larger fraction of the protein is associated with the membranes; this binding is accompanied by a large change in intrinsic fluorescence and the appearance of much greater anion permease activity. Neither membrane binding nor anion permease activity is different among isoforms. The capacity for this inserted protein to transition to the cation permease conformation is greatly decreased. We hypothesize that the previously reported change in intrinsic fluorescence as pH falls from 6 to 5 is reflecting a structural transformation to a form that is incapable of transitioning to the cation permease conformation, and differences among the isoforms have no effect on the ion permease properties of this conformation.

An additional novel observation is that calcium and magnesium ions are equally effective in supporting the cation channel activity of ApoL1. This strongly suggests that the role of divalent cation is not through some high-affinity binding by the protein, but more likely a less specific charge-bridging effect allowing negatively charged protein to interact with the negatively charged surface of phospholipid membranes.

Other investigations assessing for differences in ion channel activity of ApoL1 isoforms have been published. Thompson and Finkelstein (8) used electrophysiologic methods with planar lipid bilayer techniques to assess the channel activity of purified recombinant ApoL1, reporting no differences in channel characteristics among the isoforms. It is important to note that planar lipid bilayer methods and vesicle-based efflux assays, while both being channel assays, yield different kinds of information and can be thought of as complementary experimental systems. Lipid bilayer methods are an unparalleled approach to define single-channel properties of purified protein, but are less powerful tools to quantify specific activity, whereas vesicle efflux assays can provide quantitative data about activity of a population, but little information about single-channel properties. Thus, the observations of Thompson and Finkelstein are not inconsistent with ours, which also show no apparent differences in the intrinsic properties of the channel, but only a difference in specific activity of about twofold, a scale of difference that would be quite difficult to detect in lipid bilayer systems.

Investigations using purified protein and lipids have the virtue of revealing properties that are clearly due to the protein itself and not altered by any other components, expression kinetics, or membrane trafficking that compromise more complex systems. However, they are necessarily silent on the ultimate effect these activities have in living cells. Studies using cell-based expression systems to tease out ion channel activities of ApoL1 have been reported (reviewed in Friedman and Pollak, 2019 (3)). Olabisi *et al.* (11) expressed ApoL1 isoforms in HEK cells and assessed steady-state intracellular potassium concentration. They found lower intracellular potassium in cells expressing G1 and G2 compared with cells expressing G0. While this observation could be consistent with increased plasma membrane K permeability, the lower intracellular K could also be a nonspecific consequence of greater cytotoxic effect of the variant through any mechanism, leading to less active NaK ATPase activity and/or breakdown of plasma membrane integrity. O’Toole *et al.* (12) carefully calibrated levels of expression in HEK cells and assessed appearance of plasma membrane cation channel activity, finding no differences among the isoforms. Shah *et al.* (13) reported targeting of ApoL1 to mitochondria where the disease-associated variants induced opening of the mitochondrial permeability transition pore, consistent with recent evidence for trypanosome cytotoxicity. Whether this effect requires ion channel activity of ApoL1 is unclear. With each of these studies, due to the complexity of cell-based expression systems, it cannot be certain whether any differences noted are due to intrinsic properties of the protein or to other interacting components present in cells.

Pathogenic gain-of-function mutations typically show a dominant inheritance pattern, while the kidney disease phenotype associated with ApoL1 variants is recessive. One mechanism by which a gain-of-function mutation may be recessive is if the protein functions as a homomultimer, and the WT can exert a dominant negative effect on the activated variant. Whether ApoL1 functions as a channel as a monomer or a multimer is currently unknown.

Our data suggest that increased cation permeability at similar levels of protein expression could be contributing to cytotoxicity in podocytes or other cells. However, it may seem unlikely that a mere twofold increase in activity could convert a seemingly benign molecule into one driving disease. Nonetheless, the diseases associated with ApoL1 variants tend to be slowly progressive and subtle differences over a prolonged period of time (months to years) could conceivably have a large cumulative effect. Other potential mechanisms of ApoL1 action responsible for kidney injury have been proposed and are supported by evidence (3, 4, 5, 11, 12, 13, 14, 15, 16), including effects on apoptosis, autophagy, mitochrondrial function, intracellular membrane trafficking, and mediating effects of extracellular signaling molecules, among others. Relative importance of differences in ApoL1 channel activity described here *versus* other potential mechanisms of action in driving disease remains to be determined.

## Experimental procedures

### Expression constructs

pET-ApoWT encoding N-terminal 6His and T7 epitope tagged ApoL1 (G0 version) was previously described (9). The encoded protein is identical to that of Genbank accession #BC143038 (haplotype 150K, 228I, 255K) from amino acid 28 to 398, eliminating the signal sequence in amino acids 1–27 and with the addition of N-terminal 6-His and T7 epitope tags encoded by the pET28a plasmid. Plasmids encoding the G1 and G2 variants were generated from pET-ApoWT by substitution of the AleI to XhoI restriction fragment with fragments containing the variant sequences, synthesized by Integrated DNA Technologies, Coralville, IA, and the identity of the resulting plasmids confirmed by direct sequencing. All three constructs were identical except for the S342G and I384M amino acid substitutions in the G1 isoform and the N388-Y389 deletion in the G2 isoform. Recombinant ApoL1 isoforms were expressed in bacteria and purified as described (8) except that the S200 column buffer contained 0.03% n-dodecyl-β-D-maltoside (DDM) (Millipore-Sigma). For all sets of experiments comparing isoforms, the materials were prepared at the same time under identical conditions using the same reagents. Protein concentration was determined with the BCA Protein Assay Kit (Thermo Fisher Scientific) using bovine serum albumin standard. For total protein as in Figure 1, gels were stained with Instant Blue (Expedeon Inc). Protein in S200 buffer was flash frozen in liquid nitrogen and stored at –80 °C. Aliquots were thawed on ice and diluted in S200 buffer prior to assays.

### Ionophores

Valinomycin (Millipore Sigma) was dissolved at 50 mM in absolute ethanol and diluted to 1 mM in absolute ethanol immediately prior to use. Chloride Ionophore 1 (Millipore Sigma) was dissolved at 100 mM in 1-hexanol and diluted to 0.2 mM in absolute ethanol immediately prior to use.

### Membrane vesicles

Asolectin (soybean phosphatidyl choline type II, Sigma) was acetone-extracted as described (30), dried, and then dissolved in chloroform and stored at –20 °C. An aliquot of phospholipids in chloroform was dried under a stream of nitrogen and suspended at 20 mg/ml in 200 mM KCl, 5 mM HEPES pH 8.0 by five freeze–thaw cycles and vigorous vortexing. This suspension was stored at –20 °C. To generate KCl-loaded unilamellar vesicles of approximately 200 nm diameter, the lipid suspension was passed 15 times through a 200 nm pore polycarbonate membrane filter using a extruder chamber (Avanti Polar Lipids) and the protocol provided by the manufacturer (17, 18). Extruded vesicles were kept on ice and used within 24 h.

### Activity assays

Ion permease activity assays using membrane potential-driven KCl efflux detected with a chloride selective electrode were performed as previously described (9). Twenty microliters of ApoL1 in S200 buffer (150 mM NaCl, 10 mM Tris pH 8.3, 0.3% DDM) are added to 380 μl reaction mix comprised of 82 mM KCl, 127 mM sucrose, 42.1 mM buffer at the indicated pH (MES for pH 5–6.5, HEPES for pH 7.0–8.0), 2.63 mM Ca(gluconate)_2_, and 1.84 mg/ml extruded asolectin vesicles. The mixture is incubated at room temperature for 4 min before passage through a 3 ml Bio-Gel P-6DG (Bio-Rad) spin column equilibrated in 330 mM sucrose. The eluate is immediately added to 2 ml of 330 mM sucrose, 10^-5^ M KCl, 2.5 mM Ca(gluconate)_2_ and 10 mM buffer (MES for pH 5–6.5; HEPES for pH 7–8) as indicated, with continuous monitoring with a chloride selective electrode and recorded as described. After 20 s, voltage-driven efflux was initiated by addition of ionophore (2.5 μl of either 1 mM valinomycin or 0.2 mM Chloride Ionophore 1 in ethanol) and allowed to proceed for 30 s followed by addition of Triton X-100 to 0.1% to release remaining intravesicular chloride. Raw data in mV was converted to Cl concentration using a standard curve generated for the electrode under conditions of the assay. Initial fractional rates of chloride release were derived from 3 s during the maximal immediate response to addition of ionophore.

All activity comparisons were performed using material from simultaneous preparations of the three isoforms that had been diluted to 100 μg/ml protein in S200 buffer prior to assay. All assays used for direct comparison among the variants were performed on the same day using the same freshly prepared reagents.

### Membrane association assay

Membrane association assays were carried out separately under conditions optimal for the Cl permease activity and for the K permease activity. For conditions supporting Cl permease activity, 5 μl of 100 μg/ml protein in 10 mM Tris pH 8.2, 150 mM NaCl, 0.03% DDM was added to 95 μl of reaction mix (3.4 mg/ml asolectin vesicles (loaded with 200 mM KCl, 5 mM HEPES pH 8), 20 mM MES pH 5.0, 2.5 mM Ca(gluconate)_2_, 100 mM KCl, 100 mM sucrose) and incubated at room temperature for 1 min. The protein-to-lipid ratio in the assay is 1:680 mass/mass. The sample was diluted without pH shift by addition of 200 μl of 60 mM MES pH 5.0, 2.5 mM Ca(gluconate)_2_, 100 mM KCl, 100 mM sucrose and incubated for 20 s. One hundred microliters of 8 M urea was added, mixed, and then incubated at room temperature for 15 min. The sucrose concentration was brought to 40% by addition of 1.6 ml of 50% sucrose. The 2 ml sample was layered below 1 ml of 30% sucrose in 8 mM MES pH 5.0, which was in turn below 1.7 ml of sucrose-free 8 mM MES pH 5.0. The step gradients were centrifuged at 200,000*g* in a Beckman Coulter TLA-110 rotor for 90 min at 4° C. The vesicles were collected from the 0 to 30% sucrose interface. Equal fractions of samples were diluted 4 fold and centrifuged at 100,000*g* for 1 h. The membrane pellets were dried, separated by SDS-PAGE, blotted to polyvinylidene fluoride membrane, and probed by western blotting with antibody to the T7 epitope (MilliporeSigma product no. 69522-3) with chemiluminescence detection using a digital imaging system. Serial dilutions of purified protein were included on the gel to generate an isotype and blot-specific standard curve for each protein.

For conditions supporting K permease activity, 5 μl of 100 μg/ml protein in 10 mM Tris pH 8.2, 150 mM NaCl, 0.03% DDM was added to 95 μl of reaction mix (3.4 μg/ml asolectin vesicles (loaded with 200 mM KCl, 5 mM HEPES pH 8), 20 mM MES pH 6.0, 2.5 mM Ca(gluconate)_2_, 100 mM KCl, 100 mM sucrose) and incubated at room temperature for 1 min. The sample was diluted with pH shift to 7.5 by addition of 200 μl of 60 mM HEPES pH 8.0, 2.5 mM Ca(gluconate)_2_, 100 mM KCl, 100 mM sucrose and incubated for 20 s. One hundred microliters of 500 mM of Na_2_CO_3_ was added and incubated at room temperature for 15 min. The sucrose concentration was brought to 40% by addition of 1.6 ml of 50% sucrose. The 2 ml sample was layered below 1 ml of 30% sucrose in 8 mM Tris pH 8.3, which was in turn below 1.7 ml of sucrose-free 8 mM Tris pH 8.3. Sucrose cushions were centrifuged and sample collected and processed as above.

### Statistics

Significance of differences between pairs of means within data sets was determined using analysis of variance (ANOVA) (19). Statistical parameters were determined using function embedded in Excel. P values were calculated using https://www.socscistatistics.com/pvalues/tdistribution.aspx. All data points are presented as the mean ± standard deviation (SD), determined with the STDEV.S function of Excel for each individual group of replicas

## Data availability

All data supporting this paper will be shared upon request to Dr John C. Edwards, john.edwards@health.slu.edu.

## Conflict of interest

The authors declare that they have no conflict of interest with the contents of this article.
